# Bis(μ-2,2′-biimidazole-κ^2^ *N* ^3^:*N* ^3′^)bis­[aqua­copper(I)] sulfate

**DOI:** 10.1107/S1600536809046996

**Published:** 2009-11-14

**Authors:** Xiutang Zhang, Peihai Wei, Bo Hu, Bin Li, Congwen Shi

**Affiliations:** a Advanced Material Institute of Research, Department of Chemistry and Chemical Engineering, ShanDong Institute of Education, Jinan 250013, People’s Republic of China; b College of Chemistry and Chemical Engineering, Liaocheng University, Liaocheng 252059, People’s Republic of China

## Abstract

In the structure of the title compound, [Cu_2_(C_6_H_6_N_4_)_2_(H_2_O)_2_]SO_4_, the asymmetric unit contains half each of two 2,2′-diimidazole ligands, one Cu^+^ cation, one water mol­ecule and half of a sulfate anion (2 symmetry). The dinuclear complex is completed through a twofold rotation axis, leading to a twisted ten-membered ring mol­ecule. The dihedral angle between the two symmetry-related 2,2′-diimidazole ligands is 23.6 (1)°. The copper centre is coordinated by two N atoms of two symmetry-related 2,2′-diimidazole ligands in an almost linear fashion. The water mol­ecule exhibits a weak coordination to Cu^+^ with a more remote distance of 2.591 (2) Å. The distance between the two copper centres is 2.5956 (6) Å. O—H⋯O and N—H⋯O hydrogen bonds between the complex cation, the water mol­ecule and the sulfate anion lead to the formation of a three-dimensional network.

## Related literature

For background to metal organic framework structures, see: Lee *et al.* (2000 ▶).
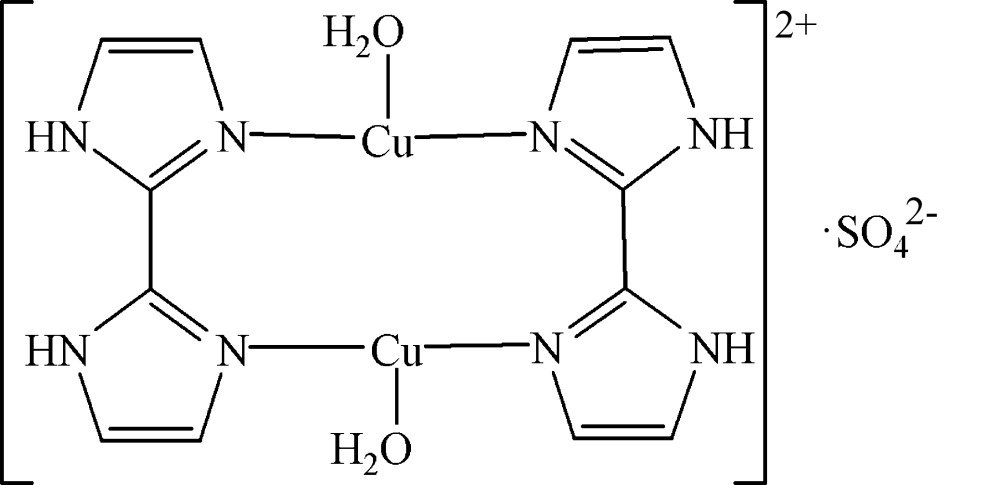


## Experimental

### 

#### Crystal data

[Cu_2_(C_6_H_6_N_4_)_2_(H_2_O)_2_]SO_4_
*M* *_r_* = 527.50
Monoclinic, 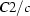

*a* = 12.7597 (7) Å
*b* = 14.8594 (7) Å
*c* = 10.6375 (5) Å
β = 114.777 (3)°
*V* = 1831.22 (16) Å^3^
*Z* = 4
Mo *K*α radiation
μ = 2.49 mm^−1^
*T* = 273 K
0.12 × 0.10 × 0.08 mm

#### Data collection

Bruker APEXII CCD diffractometer
Absorption correction: multi-scan (*SADABS*; Bruker, 2001 ▶) *T*_min_ = 0.754, *T*_max_ = 0.826
9619 measured reflections
1630 independent reflections
1522 reflections with *I* > 2σ(*I*)
*R*_int_ = 0.023

#### Refinement

*R*[*F* ^2^ > 2σ(*F* ^2^)] = 0.025
*wR*(*F* ^2^) = 0.073
*S* = 1.00
1630 reflections
148 parameters
H atoms treated by a mixture of independent and constrained refinement
Δρ_max_ = 0.30 e Å^−3^
Δρ_min_ = −0.45 e Å^−3^

### 

Data collection: *APEX2* (Bruker, 2004 ▶); cell refinement: *SAINT-Plus* (Bruker, 2001 ▶); data reduction: *SAINT-Plus*; program(s) used to solve structure: *SHELXS97* (Sheldrick, 2008 ▶); program(s) used to refine structure: *SHELXL97* (Sheldrick, 2008 ▶); molecular graphics: *SHELXTL* (Sheldrick, 2008 ▶); software used to prepare material for publication: *SHELXL97*.

## Supplementary Material

Crystal structure: contains datablocks global, I. DOI: 10.1107/S1600536809046996/wm2276sup1.cif

Structure factors: contains datablocks I. DOI: 10.1107/S1600536809046996/wm2276Isup2.hkl

Additional supplementary materials:  crystallographic information; 3D view; checkCIF report

## Figures and Tables

**Table:** 

Cu1—N4	1.8953 (18)
Cu1—N2	1.9006 (18)

**Table:** 

N4—Cu1—N2	173.20 (8)

**Table 2:** Hydrogen-bond geometry (Å, °)

*D*—H⋯*A*	*D*—H	H⋯*A*	*D*⋯*A*	*D*—H⋯*A*
O1*W*—H1*W*⋯O2^i^	0.819 (6)	2.037 (8)	2.848 (3)	170 (5)
O1*W*—H2*W*⋯O1^ii^	0.82 (3)	2.294 (14)	3.072 (4)	159 (4)
N3—H3*A*⋯O1^iii^	0.970 (14)	1.794 (13)	2.697 (3)	153 (3)
N1—H1*A*⋯O2^iv^	0.972 (15)	1.804 (9)	2.743 (3)	162 (3)

Symmetry codes: (i) 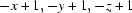; (ii) 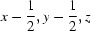; (iii) 
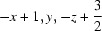
; (iv) 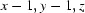.

**Fig. 1.**
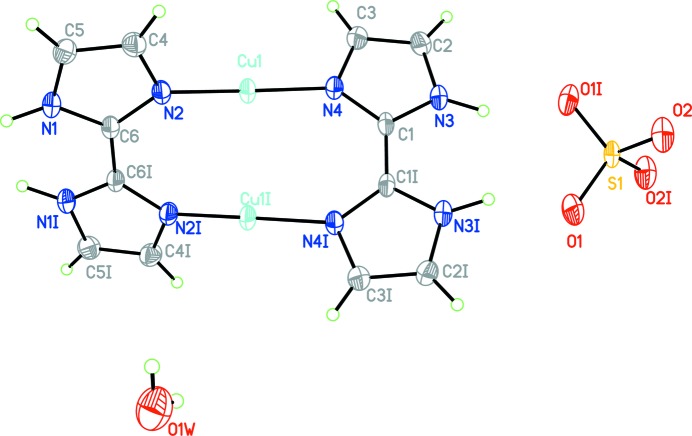
A view of the title compound with the unique atom-labelling scheme. Displacement ellipsoids are drawn at the 30% probability level. [Symmetry code: #I -x,y,-z + 3/2]

**Fig. 2.**
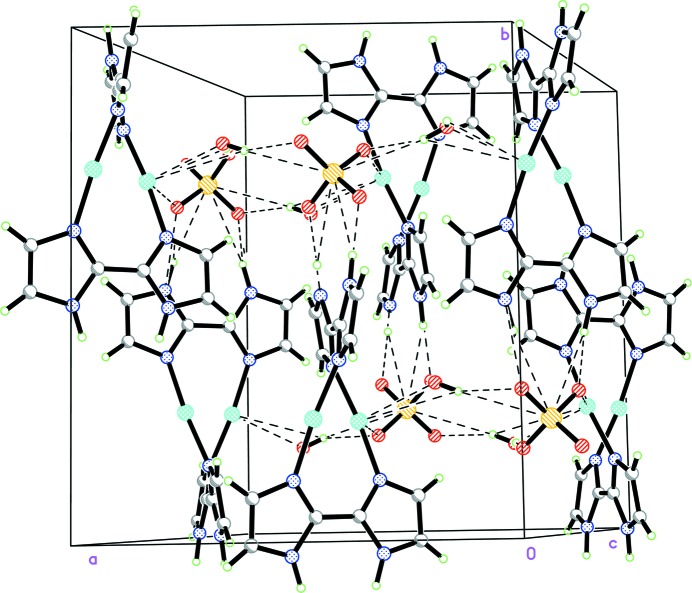
A view of the packing diagram of the title compound. Displacement ellipsoids are drawn at the 30% probability level.

## supplementary crystallographic information

### Comment

The design and synthesis of metal-organic frameworks (MOFs) has attracted continuous research interest not only because of their appealing structural and topological novelties, but also due to their optical, electronic, magnetic, and catalytic properties, as well as their potential medical applications (Lee *et al.* 2000). Here, we report the structure of the title compound.

As shown in Figure 1, the Cu^+^ cation is coordianted by two N atoms from two 2,2'-diimidazole molecules, showing an almost linear coordination to Cu(I), the Cu—N bond lengths being 1.8953 (18) and 1.9006 (18) Å, respectively. The separation between the two Cu^+^ cores is 2.5956 (6) Å. Moreover, the water molecule exhibits a weak coordination to Cu(I) with a more remote distance of 2.591 (2) Å. Each two Cu(I) ions and two 2,2'-diimidazole molecules form one ten-membered ring molecle via a twofold axis as symmetry element. The dihedral angle between two symmetry-related 2,2'-diimidazole molecules is 23.6 (1) °. In the voids of the packing, there is an intricate hydrogen bonding of the type O—H···O and N—H···O, as shown in Figure 2 and Table 2.

### Experimental

A mixture of 2,2'-diimidazole (1 mmol, 0.14 g), oxalic acid (1 mmol, 0.09 g), copper(II) sulfate pentahydrate (1 mmol, 0.25 g), and 10 ml H_2_O was heated to 443 K for one day in an autoclave. Red crystals were obtained after cooling to room temperature with a yield of 82%. Elemental Analysis. Calc. for C_12_H_16_Cu_2_N_8_O_6_S: C 27.30, H 3.03, N 21.23%; Found: C 27.15, H 2.95, N 21.11%. Under the given hydrothermal conditions, Cu(II) was apparently reduced to Cu(I), leading to the formation of the title complex.

### Refinement

All hydrogen atoms bound to carbon were refined using a riding model with C—H = 0.93 Å and *U*_iso_(H) = 1.2Ueq(C). The H atoms of the water molecule were located from difference density maps and were refined with distance restraints of d(H–H) = 1.38 (2) Å, d(O–H) = 0.88 (2) Å, and with a fixed *U*_iso_ of 0.80 Å^2^. The H atoms on nitrogen atoms were located from difference density maps and were refined with distance restraints of d(N–H) = 0.97 (2) Å.

### Crystal data

**Table:**

[Cu_2_(C_6_H_6_N_4_)_2_(H_2_O)_2_]SO_4_	*F*(000) = 1064
*M**_r_* = 527.50	*D*_x_ = 1.913 Mg m^−^^3^
Monoclinic, *C*2/*c*	Mo *K*α radiation, λ = 0.71073 Å
Hall symbol: -C 2yc	Cell parameters from 5587 reflections
*a* = 12.7597 (7) Å	θ = 0.00–0.00°
*b* = 14.8594 (7) Å	µ = 2.49 mm^−^^1^
*c* = 10.6375 (5) Å	*T* = 273 K
β = 114.777 (3)°	Block, red
*V* = 1831.22 (16) Å^3^	0.12 × 0.10 × 0.08 mm
*Z* = 4	

### Data collection

**Table:**

Bruker APEXII CCD diffractometer	1630 independent reflections
Radiation source: fine-focus sealed tube	1522 reflections with *I* > 2σ(*I*)
graphite	*R*_int_ = 0.023
φ and ω scans	θ_max_ = 25.0°, θ_min_ = 2.2°
Absorption correction: multi-scan (*SADABS*; Bruker, 2001)	*h* = −15→15
*T*_min_ = 0.754, *T*_max_ = 0.826	*k* = −17→17
9619 measured reflections	*l* = −12→12

### Refinement

**Table:**

Refinement on *F*^2^	Primary atom site location: structure-invariant direct methods
Least-squares matrix: full	Secondary atom site location: difference Fourier map
*R*[*F*^2^ > 2σ(*F*^2^)] = 0.025	Hydrogen site location: inferred from neighbouring sites
*wR*(*F*^2^) = 0.073	H atoms treated by a mixture of independent and constrained refinement
*S* = 1.00	*w* = 1/[σ^2^(*F*_o_^2^) + (0.047*P*)^2^ + 2.1669*P*] where *P* = (*F*_o_^2^ + 2*F*_c_^2^)/3
1630 reflections	(Δ/σ)_max_ = 0.008
148 parameters	Δρ_max_ = 0.30 e Å^−^^3^
0 restraints	Δρ_min_ = −0.45 e Å^−^^3^

### Special details

**Table:**

Geometry. All e.s.d.'s (except the e.s.d. in the dihedral angle between two l.s. planes) are estimated using the full covariance matrix. The cell e.s.d.'s are taken into account individually in the estimation of e.s.d.'s in distances, angles and torsion angles; correlations between e.s.d.'s in cell parameters are only used when they are defined by crystal symmetry. An approximate (isotropic) treatment of cell e.s.d.'s is used for estimating e.s.d.'s involving l.s. planes.
Refinement. Refinement of *F*^2^ against ALL reflections. The weighted *R*-factor *wR* and goodness of fit *S* are based on *F*^2^, conventional *R*-factors *R* are based on *F*, with *F* set to zero for negative *F*^2^. The threshold expression of *F*^2^ > σ(*F*^2^) is used only for calculating *R*-factors(gt) *etc*. and is not relevant to the choice of reflections for refinement. *R*-factors based on *F*^2^ are statistically about twice as large as those based on *F*, and *R*- factors based on ALL data will be even larger.

### Fractional atomic coordinates and isotropic or equivalent isotropic displacement parameters (Å^2^)

**Table:**

	*x*	*y*	*z*	*U*_iso_*/*U*_eq_	
Cu1	0.01189 (3)	0.265145 (18)	0.63467 (3)	0.04181 (14)	
S1	1.0000	0.76744 (5)	0.7500	0.0392 (2)	
C1	0.03743 (17)	0.45644 (13)	0.7144 (2)	0.0299 (4)	
C2	0.14997 (19)	0.50798 (15)	0.6214 (2)	0.0379 (5)	
H2	0.1923	0.5464	0.5913	0.045*	
C3	0.1405 (2)	0.41751 (15)	0.6049 (2)	0.0400 (5)	
H3	0.1766	0.3829	0.5616	0.048*	
C4	−0.1342 (2)	0.12062 (16)	0.4636 (2)	0.0411 (5)	
H4	−0.1675	0.1563	0.3848	0.049*	
C5	−0.1485 (2)	0.03051 (16)	0.4682 (2)	0.0410 (5)	
H5	−0.1917	−0.0068	0.3944	0.049*	
C6	−0.03621 (17)	0.07952 (13)	0.6765 (2)	0.0302 (4)	
N1	−0.08656 (15)	0.00532 (12)	0.6033 (2)	0.0352 (4)	
N2	−0.06269 (16)	0.15125 (12)	0.59369 (19)	0.0356 (4)	
N3	0.08523 (15)	0.53162 (11)	0.69092 (19)	0.0338 (4)	
N4	0.06894 (16)	0.38469 (12)	0.66225 (19)	0.0350 (4)	
O1	0.9542 (2)	0.71052 (14)	0.8273 (3)	0.0695 (6)	
O2	0.90665 (16)	0.82506 (12)	0.6552 (2)	0.0561 (5)	
O1W	0.1960 (2)	0.18900 (17)	0.6389 (3)	0.0708 (6)	
H1W	0.169 (3)	0.191 (4)	0.5542 (3)	0.13 (2)*	
H2W	0.2621 (13)	0.208 (3)	0.678 (3)	0.082 (12)*	
H3A	0.069 (2)	0.5926 (7)	0.710 (3)	0.061 (8)*	
H1A	−0.079 (2)	−0.0551 (7)	0.641 (3)	0.060 (8)*	

### Atomic displacement parameters (Å^2^)

**Table:**

	*U*^11^	*U*^22^	*U*^33^	*U*^12^	*U*^13^	*U*^23^
Cu1	0.0621 (2)	0.01984 (19)	0.0446 (2)	−0.00492 (11)	0.02347 (16)	−0.00188 (10)
S1	0.0481 (5)	0.0179 (4)	0.0512 (5)	0.000	0.0202 (4)	0.000
C1	0.0328 (10)	0.0188 (9)	0.0321 (10)	0.0000 (8)	0.0075 (8)	0.0009 (8)
C2	0.0378 (11)	0.0326 (11)	0.0440 (12)	−0.0024 (9)	0.0179 (10)	0.0032 (9)
C3	0.0457 (12)	0.0332 (12)	0.0448 (13)	0.0033 (10)	0.0227 (10)	0.0008 (10)
C4	0.0462 (12)	0.0379 (12)	0.0345 (11)	−0.0033 (10)	0.0122 (10)	0.0019 (10)
C5	0.0424 (12)	0.0387 (13)	0.0383 (12)	−0.0091 (10)	0.0133 (10)	−0.0073 (10)
C6	0.0317 (10)	0.0214 (10)	0.0384 (11)	−0.0013 (8)	0.0157 (8)	−0.0008 (8)
N1	0.0373 (9)	0.0235 (9)	0.0432 (10)	−0.0028 (7)	0.0153 (8)	−0.0024 (8)
N2	0.0438 (10)	0.0245 (9)	0.0368 (10)	−0.0024 (7)	0.0154 (8)	0.0006 (7)
N3	0.0350 (9)	0.0208 (9)	0.0417 (10)	−0.0009 (7)	0.0124 (8)	0.0009 (7)
N4	0.0436 (10)	0.0221 (9)	0.0391 (10)	0.0003 (7)	0.0173 (8)	−0.0002 (7)
O1	0.1064 (17)	0.0292 (9)	0.0917 (16)	−0.0109 (11)	0.0600 (14)	0.0037 (10)
O2	0.0531 (10)	0.0340 (9)	0.0671 (12)	0.0030 (8)	0.0113 (9)	0.0031 (8)
O1W	0.0653 (14)	0.0608 (14)	0.0795 (17)	−0.0082 (11)	0.0236 (12)	−0.0130 (12)

### Geometric parameters (Å, °)

**Table:**

Cu1—N4	1.8953 (18)	C3—H3	0.9300
Cu1—N2	1.9006 (18)	C4—C5	1.355 (3)
Cu1—Cu1^i^	2.5956 (6)	C4—N2	1.377 (3)
S1—O1	1.462 (2)	C4—H4	0.9300
S1—O1^ii^	1.462 (2)	C5—N1	1.370 (3)
S1—O2^ii^	1.4704 (18)	C5—H5	0.9300
S1—O2	1.4704 (18)	C6—N2	1.333 (3)
C1—N4	1.339 (3)	C6—N1	1.347 (3)
C1—N3	1.345 (3)	C6—C6^i^	1.446 (4)
C1—C1^i^	1.447 (4)	N1—H1A	0.972 (15)
C2—C3	1.355 (3)	N3—H3A	0.970 (14)
C2—N3	1.366 (3)	O1W—H1W	0.819 (6)
C2—H2	0.9300	O1W—H2W	0.82 (3)
C3—N4	1.383 (3)		
			
N4—Cu1—N2	173.20 (8)	N2—C4—H4	125.3
N4—Cu1—Cu1^i^	92.47 (6)	C4—C5—N1	106.3 (2)
N2—Cu1—Cu1^i^	88.35 (6)	C4—C5—H5	126.9
O1—S1—O1^ii^	109.29 (18)	N1—C5—H5	126.8
O1—S1—O2^ii^	110.56 (13)	N2—C6—N1	110.22 (19)
O1^ii^—S1—O2^ii^	108.83 (13)	N2—C6—C6^i^	125.81 (12)
O1—S1—O2	108.83 (13)	N1—C6—C6^i^	123.98 (13)
O1^ii^—S1—O2	110.56 (13)	C6—N1—C5	107.94 (18)
O2^ii^—S1—O2	108.77 (15)	C6—N1—H1A	125.3 (18)
N4—C1—N3	110.28 (19)	C5—N1—H1A	126.8 (18)
N4—C1—C1^i^	126.55 (12)	C6—N2—C4	106.10 (18)
N3—C1—C1^i^	123.17 (12)	C6—N2—Cu1	126.64 (15)
C3—C2—N3	106.5 (2)	C4—N2—Cu1	125.59 (15)
C3—C2—H2	126.7	C1—N3—C2	108.11 (18)
N3—C2—H2	126.7	C1—N3—H3A	125.6 (17)
C2—C3—N4	109.4 (2)	C2—N3—H3A	125.9 (17)
C2—C3—H3	125.3	C1—N4—C3	105.68 (18)
N4—C3—H3	125.3	C1—N4—Cu1	130.40 (15)
C5—C4—N2	109.4 (2)	C3—N4—Cu1	122.96 (15)
C5—C4—H4	125.3	H1W—O1W—H2W	115 (4)

Symmetry codes: (i) −*x*, *y*, −*z*+3/2; (ii) −*x*+2, *y*, −*z*+3/2.

### Hydrogen-bond geometry (Å, °)

**Table:**

*D*—H···*A*	*D*—H	H···*A*	*D*···*A*	*D*—H···*A*
O1W—H1W···O2^iii^	0.82 (1)	2.04 (1)	2.848 (3)	170 (5)
O1W—H2W···O1^iv^	0.82 (3)	2.29 (1)	3.072 (4)	159 (4)
N3—H3A···O1^v^	0.97 (1)	1.79 (1)	2.697 (3)	153 (3)
N1—H1A···O2^vi^	0.97 (2)	1.80 (1)	2.743 (3)	162 (3)

Symmetry codes: (iii) −*x*+1, −*y*+1, −*z*+1; (iv) *x*−1/2, *y*−1/2, *z*; (v) −*x*+1, *y*, −*z*+3/2; (vi) *x*−1, *y*−1, *z*.
